# SPOUSAL ASSOCIATIONS IN MONTHLY REPORTS OF DISABILITY IN THE PRECIPITATING EVENTS PROJECT

**DOI:** 10.1093/geroni/igz038.2484

**Published:** 2019-11-08

**Authors:** Joan K Monin, Holly Laws, Evelyne Gahbauer, Terrence E Murphy, Thomas M Gill

**Affiliations:** 1 Yale School of Public Health, New Haven, Connecticut, United States; 2 University of Massachusetts Amherst, Amherst, Massachusetts, United States; 3 Yale School of Medicine, New Haven, Connecticut, United States

## Abstract

While many prior studies have evaluated the antecedents and consequences of changes in disability, few have considered the social context. As nearly 60% of older adults currently live with a spouse or intimate partner, it is important to examine spousal influences on disability. This study examined spousal associations in self-reported disability using data from the Precipitating Events Project, an ongoing longitudinal study of 754 initially nondisabled community living adults age 70 and over who have had monthly assessments of functional status since 1999. We hypothesized that one spouse’s level of disability would be associated with increases in the other spouse’s subsequent disability. We used the Actor Partner Interdependence Model (APIM), a statistical modeling framework that accounts for the interdependence in two-person data and tests the associations of both self (actor) and partner influences on outcomes. We used multilevel, longitudinal APIMs to examine lagged associations in spouses’ monthly reports of disability in 13 activities of daily living (e.g., walking a quarter mile, bathing) in the 37 married couples. As hypothesized, one partner’s prior disability level was significantly associated with the other partner’s (the actor’s) subsequent disability level (B = .674, SE = .012, p < .001) after controlling for the actor’s prior disability level. Also, when both couple members had higher levels of prior disability, they were particularly at risk of subsequent increases in disability (B = .016, SE = .003, p < .001). Incorporating partner disability level in modeling individuals’ outcomes provides greater precision in predicting future disability levels.

